# Assessing the risk of ASFV entry into Japan through pork products illegally brought in by air passengers from China and fed to pigs in Japan

**DOI:** 10.1371/journal.pone.0232132

**Published:** 2020-05-05

**Authors:** Katsuaki Sugiura, Zhihao Lei, Caitlin Holley, Takeshi Haga

**Affiliations:** 1 Department of Global Agricultural Sciences, Graduate School of Agricultural and Life Sciences, The University of Tokyo, Tokyo, Japan; 2 Regional Representation for Asian and Pacific, World Organisation for Animal Health (OIE), Bunkyo-ku, Tokyo, Japan; 3 Department of Veterinary Medical Sciences, Graduate School of Agricultural and Life Sciences, The University of Tokyo, Tokyo, Japan; Panstwowy Instytut Weterynaryjny - Panstwowy Instytut Badawczy w Pulawach, POLAND

## Abstract

A risk assessment was conducted to assess the risk of ASFV entry into Japan through pork products illegally brought in by air passengers from China and fed to pigs in Japan. Scenario tree modelling was used with the following entry and exposure pathway considered to be the most likely route of ASF entry: an ASFV infected pork product is illegally brought into Japan by air travellers from China; this pork product is then used in a restaurant where scrap waste is recycled for animal feed and subsequently fed to pigs without being heat-treated. Input parameter values were based on surveys conducted by the authors, scientific data gathered from the literature and official data published by government agencies. The annual probability of ASFV entry into Japan via this pathway was predicted to be 0.20 (90% prediction interval: 0.00–0.90). The wide prediction interval was mainly caused by the uncertainty regarding the dose response relation of ASFV, followed by the probability of an ASF infected pig dying on affected farms, the loading of ASFV in an infected pig and the probability of an illegally imported pork product being heat-treated in China and used in restaurants. The results of scenario analysis revealed that the annual probability of ASFV entry into Japan will increase with an increase in the number of ASF affected farms in China. The probability of ASFV entry will increase substantially even if only a small proportion of Ecofeed is not heat-treated during the production process. The probability will decrease if an increased proportion of farms that feed swill apply heat-treatment before feeding swill to their pigs. These findings indicate that stringent application of heat-treatment of Ecofeed and swill is key to protecting the Japanese pig industry from the introduction of ASFV.

## Introduction

African swine fever (ASF) is a highly contagious disease affecting pigs. It is caused by African swine fever virus (ASFV), which belongs to the genus Asfivirus of the Asfaviridae family [1]. ASF can spread through direct or indirect contact and causes high mortality. The ASFV persists for a long time in the environment a in a variety of pig products. Wild boar can harbor the virus and ASF may become endemic with or without an added transmission cycle through Ornithodoros ticks [2].

Traditionally ASF was confined to Africa, with occasional incursions into other regions until in 2007 when ASF was introduced into Georgia, a Caucasus country [3]. From Georgia, the disease spread to neighboring countries, including Armenia, the Russian Federation in 2007, and Azerbaijan in 2008. From the Caucasus, the disease continued northward and westward in Ukraine and Belarus in 2013, in Poland, Latvia, Estonia and Lithuania in 2014, and in Czech Republic and Romania in 2017, with additional outbreaks in the east of the Russian Federation in domestic pig and wild boar populations [3, 4]. In March 2017, ASF was reported in Irkutsk, Russian Federation, approx. 1,000 km from the border with China [5].

ASF appeared in China, the world’s largest pig producer and pig meat consumer, with the first outbreak reported on a farm near Shenyang City in Liaoning Province on 3 August 2018 [6, 7]. The pigs on this farm had been fed table scraps and developed acute clinical disease around mid-June 2018 [7]. Since then, ASF spread in China, with 163 outbreaks reported in 22 provinces, 5 autonomous regions and 5 municipalities as of 16 January 2020 [8]. The epidemic of ASF in China constitutes an important threat to Japan. The outbreaks reported in Vietnam, Mongolia, Cambodia, Hong Kong SAR, Democratic People’s Republic of Korea, Laos, Myanmar, the Philippines, Republic of Korea, Timor-Leste and Indonesia at the time of writing of this paper further highlights the risk of spread of ASF to Japan [8].

Japan has a geographical advantage of being isolated by the sea and thus the risk of introduction of ASF through importation of live pigs or pork products by land transportation and movements of wild boar is practically null. However, Japan is subject to considerable international movements of aircrafts carrying increasing numbers of travelers: a total of 26.3 million foreign visitors traveled to Japan by aircraft in 2018, accounting for 92% of all foreign visitors. Of this, 5.8 million were from China, accounting for 22% of the foreign air travelers [9].

The increasing number of travelers coming to Japan might also increase the number of incidences of illegal importation of pig products into Japan, thus increasing the probability of introduction of ASF into Japan. In 2018, 42,280 cases of illegal importation of pork products by Chinese travelers were detected by detector dogs and/or interrogation by the customs officers at the airports, accounting for 45% of all confiscated products [10].

In response to the increasing risk of introduction of ASF, the Japanese government has tightened quarantine operations at airports and seaports, especially for travelers from China, by

- strengthening measures and penalties against illegal importation of meat and meat products by air passengers [11];
- raising the awareness by the use of posters and inflight announcements [12];
- stringent application of penalties for travelers attempting to illegally import meat and meat products [12]; and
- increasing the number of detector dogs at the airports [13].

The possibility of animal products infected with ASFV being introduced into Japan has been identified previously as one of the highest risks for the entry of ASF into Japan [14]. Given the importance of the pig industry in Japan and the current situation of ASF in China, this study aimed at assessing the risk of ASFV entry into Japan through pig products illegally brought in by air passengers from China and fed to pigs in Japan through swill feeding.

## Materials and methods

### Model development

#### Model structure

**Fig 1:**
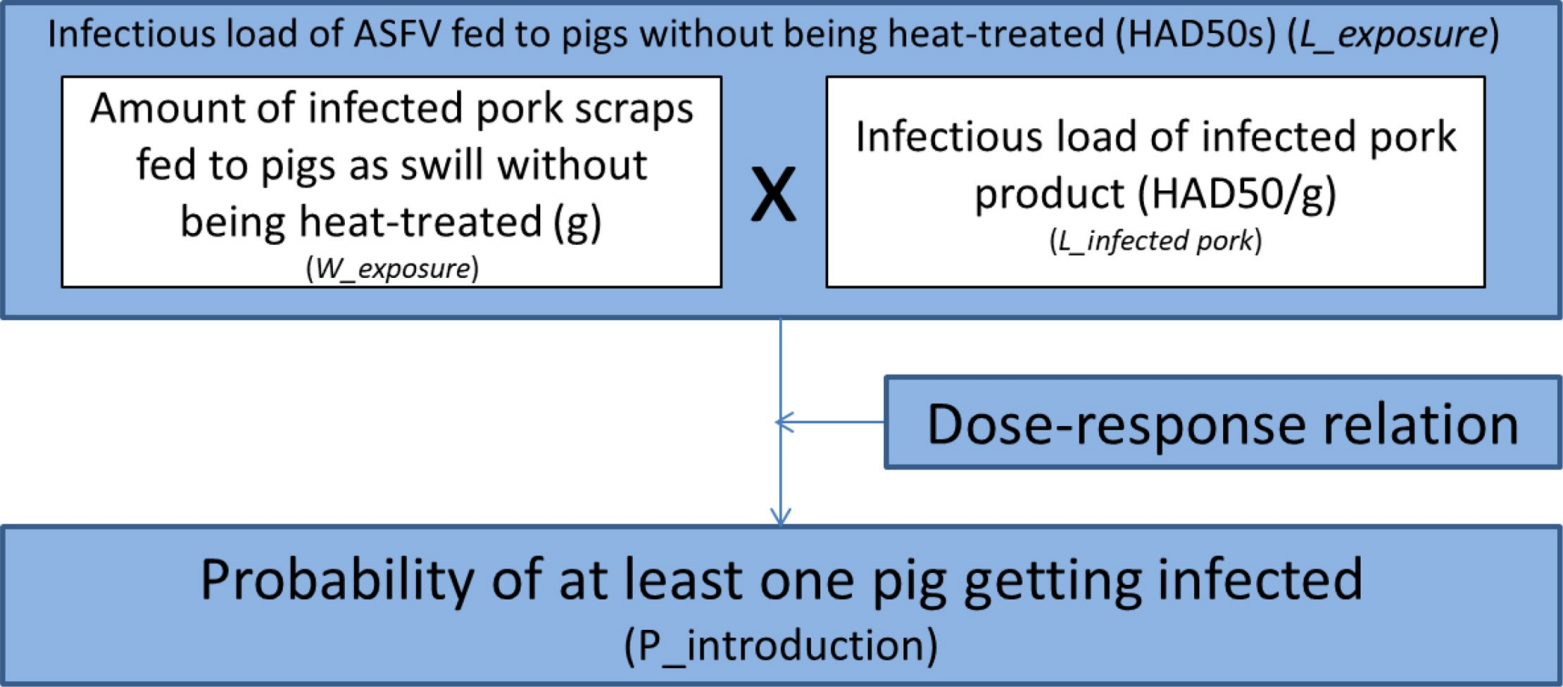
Model structure for assessing the probability of ASF introduction.

**Fig 2:**
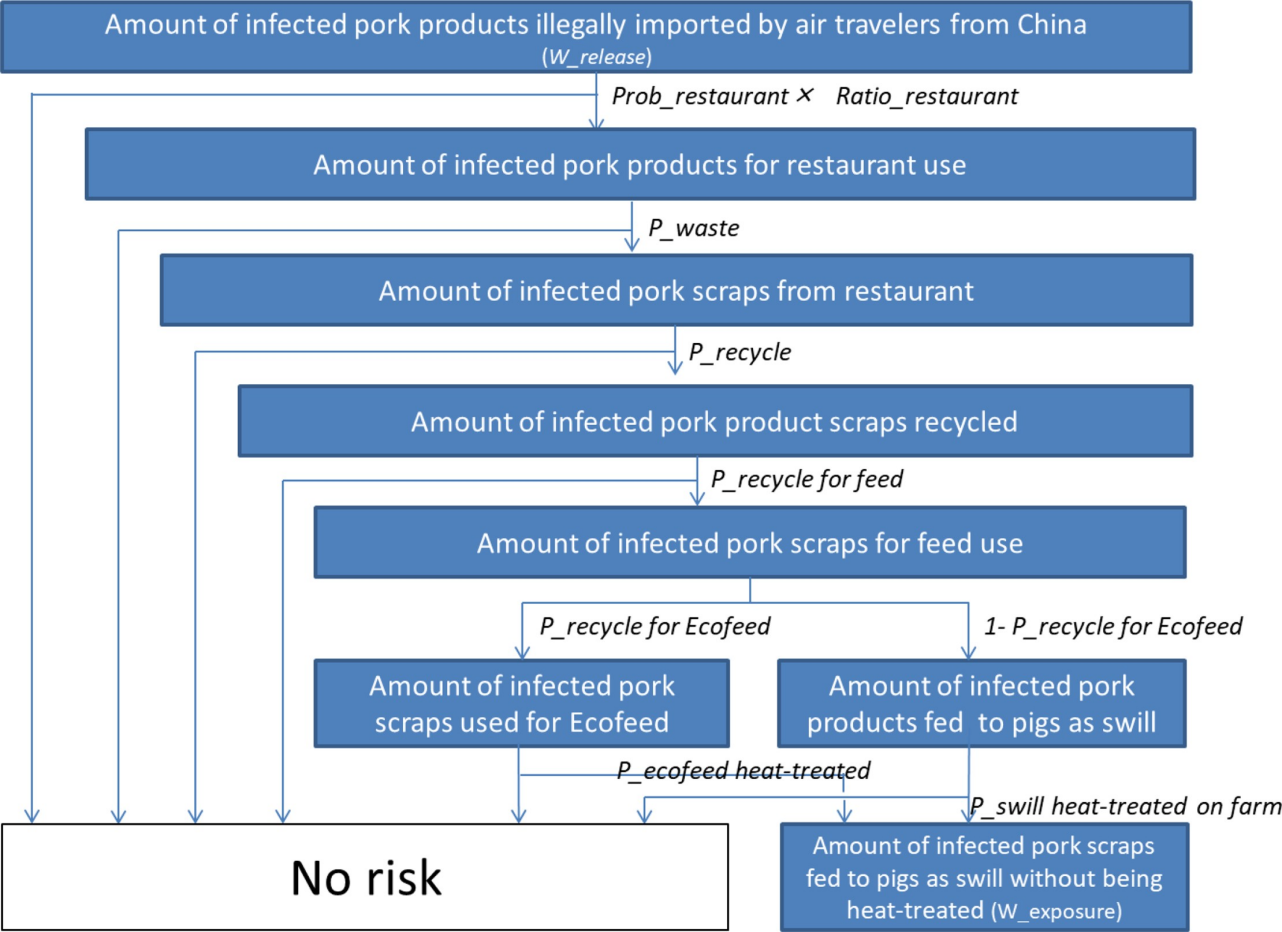
Model structure for estimating the amount of infected pork scraps fed to pigs as swill without being heat-treated (*W_exposure*).

We developed a quantitative stochastic risk assessment model. The model structure is shown in Figs 1 and 2. First we estimated the total amount of infected pig products illegally imported by air travelers from China into Japan (*W_release*). From this, we then estimated the amount of infected pork scraps originating from pork products illegally imported by air travelers from China and being fed to pigs in Japan as swill without being heat-treated (*W_exposure*). We multiplied this with the infectious load of infected pork product (*L_infected pork*), to estimate the infectious load of ASFV fed to pigs without being heat-treated (*L_exposure*). Using this load and dose response relation, we assessed the risk of at least one pig becoming infected with ASFV (*P_introduction*).

#### Total amount of infected pig products illegally imported by air travelers from China into Japan (g) (*W_release*)

We assumed that infected pig products are brought from China into Japan by air travelers when:

- the pig used for the production of pork products in China is infected with ASFV;
- the pork products derived from infected pig are not heat-treated in China and
- the pork products are illegally brought in by air travelers from China.

Based on this assumption, the *W_release* can be modelled:

$$\begin{array}{l} W\_release=P\_slaughter\times \left(1‑P\_\;heat‑treated\;in\;China\right)\times N\_air\;travelers\times P\_illegal\\ importation\times W\_pork\;product\;per\;traveler \end{array}$$ Model 1

where *P_slaughter* is the prevalence of ASF infected pigs slaughtered for consumption in China; *P_heat-treated in China* is the probability that the pork product brought in illegally is heat-treated in China; *N_air travelers* is the number of air travelers that visit Japan annually; *P_illegal importation* is the probability that an air traveler from China illegally brings in pork products; and *W_pork product per traveler* is the average amount of pork product illegally brought in by air travelers from China.

Of these variables, *P_slaughter* was estimated using the model developed by VLA [15]:

$$P\_slaughter=\frac{N\_affected\_farms\times H\times t\times (1-d)}{N\_pigs\times P\_notfification}$$

where *P_affected farms* is the number of affected farms per year; *H* is the average herd size; *t* is the duration of infection in years; *d* is the probability an infected animal dies; *N_pigs* is the pig population in China; and *P_notification* is the probability of notification.

#### Amount of infected pork waste originating from pork products illegally brought in by air travelers from China and fed to pigs in Japan as swill without being heat-treated (g) (*W_exposure*)

We assumed that pigs in Japan are exposed to infected pig products illegally imported by air travelers when:

- the infected pig products illegally brought in were used in restaurants;
- the infected pork products are not consumed in restaurants;
- the waste from the restaurants is recycled for feed; and
- the recycled waste is used for production of Ecofeed without being properly heat-treated or
- the recycled waste is fed to pigs without being heat-treated.

Based on this assumption, *W_exposure* can be modelled:

$$\begin{array}{l} W\_exposure=W\_release\times P\_restaurant\;use\times P\_waste\times P\_recycle\times P\_recycle\;for\;feed\times \\ (P\_recycle\;for\;ecofeed\times (1‑P\_ecofeed\;heat‑treated)+(1‑P\_recycle\;for\;ecofeed)\times \\ \left(1‑P\_swill\;heat‑treated\;on\;farm)\right) \end{array}$$ Model 2

where *P_restaurant use* is the proportion of *W_release* used at restaurants*; P_waste* is the proportion of the pork product waste to the pork products used in the restaurants; *P_recycle* is the proportion of the amount of pork product waste recycled to the amount of the pork product waste produced in restaurants; *P_recycle for feed* is the proportion of recycled pork product waste used for feed to the amount of pork product waste recycled; *P_recycle for ecofeed* is the proportion of the amount of pork product waste recycled for Ecofeed to the amount of pork product waste recycle for feed; *P_ecofeed heat-treated* is the probability that pork product waste recycled for Ecofeed is heat-treated; and *P_swill heat-treated on farm* is the probability that pork product waste recycled for feed and used as swill is heat-treated before being fed to pigs.

Ecofeed is feed produced from food waste under the Law for the Promotion of Recycling of Food Waste (Law No.116, 2000) to increase the self-sufficiency of feed ingredients. There are 367 registered Ecofeed plants as of May 2017 with an annual production of 1,220,000 metric tons in terms of TDN [16].

*P_restaurant use* was modeled as:

P_restautrantuse=Prob_restaurant×Ratio_restaurant,

where *Prob_restaurant* is the probability that the pork product illegally imported is used for restaurant; and *Ratio_restaurant* is the ratio between the average weight of pork products destined for restaurants and the average weight of pork products brought in by air travelers.

In our model, we assumed that infected pork products that is consumed personally in households will present no risk. This is a reasonable assumption as food waste from Japanese households is incinerated without being recycled for other uses including use as animal feed [17].

#### Infectious load of ASFV fed to pigs without being heat-treated (*L_exposure*)

L_exposure was calculated as the product of

L_exposure=W_exposure×L_infectedpork Model 3

where *L_infected pork* is the loading of ASFV in an pig infected with ASF.

#### Probability that a pig becomes infected (*P_infection*)

Probability of a pig becoming infected through consumption of infected pork product waste (*P_infection*) was calculated using an exponential dose-response curve based on the dose-response data by McVicar [18].

P_infection=1‑exp(‑c×L_exposure/n) Model 4

where *c* is a dose reponse coefficient; and *n* is the number of pigs that consume infected pork products without being heat-treated.

#### Risk of at least one pig becoming infected (*P_introduction*)

P_introduction was calculated by:

$$P\_introduction=1‑{\left(1‑P\_infection\right)}^{n}$$ Model 5

Inserting Model 4 into Model 5,

$$\begin{array}{l} P\_introduction=1‑(1‑(1‑exp(‑c\times L\_exposure/n){)}^{n}\\ \;=1‑exp(‑c\times L\_exposure) \end{array}$$ Model 6

Thus, the variable *n* disappears, i.e. *P_introduction* does not depend on the value of *n*.

### Input variables

List of input variables used in this study is shown in Table 1.

**Table 1:** An alphabetical list of variables used in the model.

Variables	Description
*L_exposure*	Infectious load of ASFV fed to pigs without being heat-treated (HAD50s)
*L_infected pork*	Infectious load of infected pork product (HAD50s) in an ASF infected pig
*N_air travelers*	Number of air travelers that visit Japan annually
*N_pigs*	Pig population in China
*P_affected farms*	Number of affected farms per year
*P_ecofeed heat-treated*	Probability that pork product waste recycled for Ecofeed is heat-treated
*P_heat-treated in China*	Probability that the pork product brought in illegally is heat-treated in China
*P_illegal importation*	Probability that an air traveler from China illegally brings in pork products
*P_infection*	Probability of a pig becoming infected through consumption of infected pork product waste
*P_introduction*	Probability of at least one pig becoming infected
*P_notification*	Probability of notification
*P_recycle*	Proportion of the amount of pork product waste recycled to the amount of the pork product waste produced in restaurants
*P_recycle for ecofeed*	Proportion of the amount of pork product waste recycled for Ecofeed to the amount of pork product waste recycle for feed
*P_recycle for feed*	Proportion of recycled pork product waste used for feed to the amount of pork product waste recycled
*P_restaurant use*	Proportion of *W_release* used in restaurants
*P_slaughter*	Prevalence of ASF infected pigs shipped for slaughter in China
*P_swill heat-treated on farm*	Probability that pork product waste recycled for feed used as swill is heat-treated before being fed to pigs
*P_waste*	Proportion of the pork product waste to the pork products used in restaurants
*Prob_restaurant*	Probability that the pork product illegally imported is used in restaurants
*Ratio_restaurant*	Ratio between the average weight of pork products destined for restaurants and the average weight of pork products brought in by air travelers
*W_exposure*	Amount of infected pork scraps originating from pork products illegally brought in by air travelers from China and fed to pigs in Japan as swill without being heat-treated (g)
*W_pork product per traveler*	Average amount of pork product illegally brought in by air travelers from China (g)
*W_release*	Total amount of infected pork products illegally imported by air travelers from China into Japan (g)
*c*	Dose response coefficient
*d*	Probability an infected animal dies
*H*	Average herd size of pig farms in China
*n*	Number of pigs that are fed with infected pork products without being heat-treated
*t*	Duration of infection (year)

#### Number of ASF affected farms in China per year (*P_affected farms*)

*N_affected farms* is estimated to be 56 based on the number of outbreaks reported to the OIE by the Chinese government during the year 2019: during this period 57 outbreaks were reported [19], with one only involving wild boar.

#### Average pig herd size (*H*)

Because there is no official information available regarding the average pig herd size in China, *H* was assumed to be 1,644 based on the number of susceptible animals that were killed or died on ASF affected farms reported to the OIE during 2019 [19].

#### Duration of infection in years (*t*)

*t* was assumed to be 230/365 = 0.63 as assumed by VLA [15].

#### Probability an infected animal dies (*d*)

*d* was assumed to be Uniform (0.5, 1) as assumed by VLA [15].

#### Pig population in China (*N_pigs*)

*N_pigs* was estimated to be 441,589,000 based on China Animal Husbandry Yearbook 2018 [20].

#### Probability of notification (*P_notification*)

There is no official information available on the level of underreporting of ASF outbreaks in China. Under the Chinese compensation system for ASF affected farms provincial authorities are responsible for compensation and if there are insufficient funds or for other reasons, there is a chance outbreaks are not reported [21]. *P_notification* was assumed to be 0.4 as assumed by VLA [15] with some uncertainty and modeled as Pert(0.2, 0.4, 0.6).

#### Probability that the pork product brought in illegally is heat-treated in China (*P_heat-treated in China*)

*P_heat-treated in China* was modeled as Beta (69–2+1, 2+1) based on the result of testing of pork products illegally imported by air travelers from China and confiscated by Japanese Animal Quarantine Service (AQS) from 1 October 2018 to 18 February 2020: 69 products tested ASF positive with PCR, indicating that they are made from ASF infected pigs. From two of these PCR positive products, ASFV was isolated, suggesting that they were not properly heat-treated during the processing procedure in China [22].

#### Number of air travelers from China that visit Japan annually (*N_air travelers*)

*N_air travelers* was estimated to be 5,952,745 based on the Immigration Statistics 2018 issued by the Ministry of Justice [9].

#### Probability that an air traveler from China illegally brings in pork products (*P_illegal importation*)

*P_illegal importation* was modelled as Beta (7+1, 248–7+1), based on the result of a survey conducted in 2019: of 248 selected air travelers arriving in Japan from China by air, seven had illegally brought in pork products [23].

#### Average amount of pork product illegally brought in by air travelers from China (*W_pork product per traveler*)

**Fig 3:**
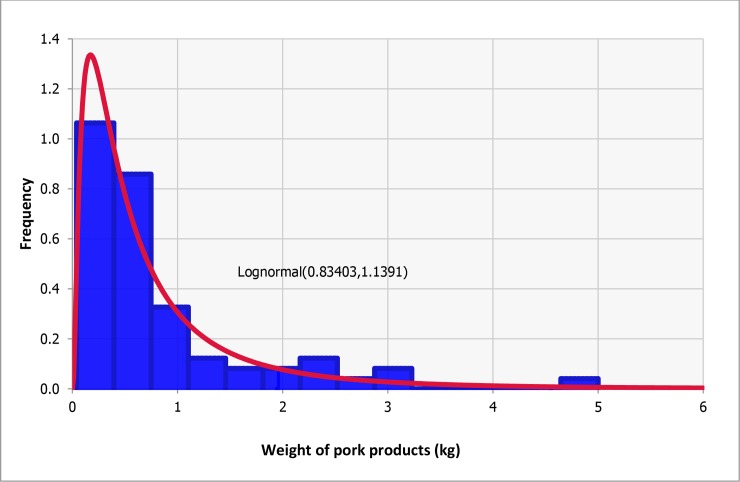
Frequency distribution of the weight of pork products tested positive by the Japanese Animal Quarantine Service during the period from 1 October to 18 February 2020 (in blue color), fitted with a Lognormal distribution (in red color) with a mean of 834g and a standard deviation of 1,139g.

The weight of pork products brought in illegally by traveler (g) was modeled as Lognormal (834, 1,139), based on the distribution of weights of the 69 pork products confiscated and tested positive for ASF by AQS [22]. *W_pork product per traveler* was estimated to be 834g by taking the average of this weight distribution (Fig 3).

#### Probability that the pork product illegally imported is used for restaurant (*Prob_restaurant*)

*Prob_restaurant use* was modeled as Beta (5+1, 69–5+1) based on the data on the weights of 69 pork products confiscated and tested positive for ASF by AQS [22]: of the 69 pork products tested PCR positive, five weighed over 2kg and were assumed to be destined for restaurant use [22]. This is based on the assumption that pork products weighing more than 2kg would be used in restaurants and those weighing less than 2kg for personal consumption [15].

#### Ratio between the average weight of pork products destined for restaurants and the average weight of pork products brought in by air travelers (*Ratio_restaurant*)

*Ratio_restaurant* was estimated to be 4.27 by taking the ratio between the average of Lognormal (834, 1,139) and the average of the fraction over 2kg of this distribution (3,560g). This is based on the assumption that pork products weighing more than 2kg are used in restaurants and those weighing less than 2kg for personal consumption [15].

#### Proportion of the pork product waste to the pork products used in the restaurants (*P_waste*)

*P_waste* was estimated to be 0.013 based on the results of a survey by the Ministry of Agriculture, Forestry and Fisheries (MAFF) [24]. According to the results of this survey 1.3% of meat and meat products used in restaurants are discarded as waste.

#### Proportion of the amount of pork product waste recycled to the amount of the pork product waste produced in restaurants (*P_recycle*)

*P_recycle* was modeled as Normal (0.2087, 0.2087×0.049) based on the results of a survey by the MAFF [24]. According to the results of this survey, 20.87% of food waste from restaurants was recycled for some use, with a relative precision of 4.9%.

#### Proportion of recycled pork product waste used for feed to the amount of pork product waste recycled (*P_recycle for feed*)

*P_recycle for feed* was estimated to be 0.2118 based on the results of a survey by the MAFF [24]. According to the results of this survey 21.18% of food waste from restaurants recycled was used for animal feed.

#### Proportion of the amount of recycled pork products used for production of Ecofeed to the amount of pork product waste recycled for feed (*P_recycle for ecofeed*)

*P_recycled for ecofeed* was estimated to be 0.929 based on data from survey results of 114 selected pig farms in Japan conducted by the Japan Pig Producers Association in 2018 [25]. According to the results of this survey, 88.9% of feed materials used in pig farms in 2018 was commercial compound feed, 1.2% rice, 9.2% Ecofeed and 0.7% other materials. We assumed that the latter two are of materials of food waste origin.

#### Probability that pork product waste recycled for feed used the production of Ecofeed is heat-treated (*P_ecofeed heat-treated*)

*P_ecofeed heat-treated* was assumed to be 1 in our baseline model, because according to the guidelines issued by the Food and Agricultural Materials Inspection Center in 2006, the heat-treatment condition of either 70 degree Celsius for 30 minutes or 80 degree Celsius for 3 minutes is required in the production of Ecofeed [26]. This heat-treatment condition meets the recommended criteria for inactivation of ASFV [26].

#### Probability that pork product waste recycled for feed used as swill is heat-treated before being fed to pigs (*P_swill heat-treated on farm*)

*P_swill heat-treated on farm* was modelled as Beta (7+1, 12–7+1). According to a questionnaire survey of 578 pig farmers who are members of JPPA in Japan conducted by the authors in 2018, 12 farmers were feeding swill containing meat products, of which seven were practicing heat-treatment before feeding swill [27].

#### Loading of ASFV in an pig infected with ASF (*L_infected pork*)

We assumed that pork products from infected pigs have a virus loading equivalent to that of muscle from an infected pig. This was modeled as 10^Uniform (3.8, 5.0) HAD50s based on the findings by Petrini et al. (2019) [28]. The ASFV infectivity titers of different muscles of infected pigs at slaughter were between 10^3.8–10^5.0 HAD50s per ml or gram [28].

#### Coefficient for the exponential dose response curve (*c*)

**Fig 4:**
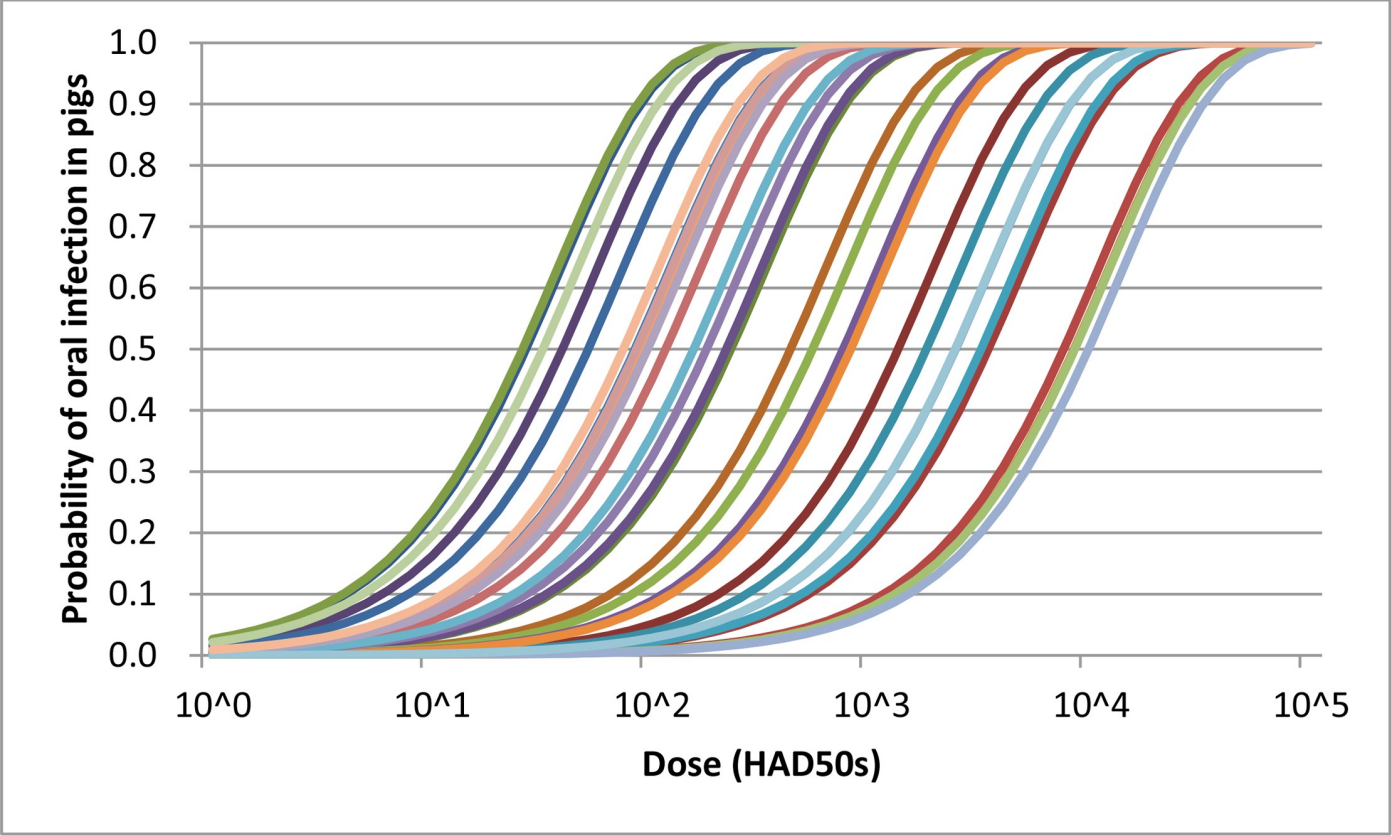
Dose response curves depicting the model *P_infection* = 1-exp(-*c* x dose (HAD50s)), where *c* = 10^Uuniform (-4.155, -1.558). This dose response model assumes any doses between 25 HAD50s and 10^3 HAD50s as a median infectious dose. 30 dose response curves randomly selected from the model are shown to provide a visual image of the distribution.

Many studies have been conducted to determine the infectious dose of ASFV. Early studies indicated a minimum dose of 10^5 HAD50s of ASFV KWH/12 was required to cause infection when administered orally in milk [29]. More recently, Howey et al. (2013) demonstrated, based on an inoculation experiment using a highly virulent Malawi strain, that 10^2 HAD50s did not induce infection but 10^4 HAD50s and 10^5 HAD50s were sufficient to cause infection in 100% of the pigs [30]. Pietschmann et al. (2015) demonstrated that as low as 3 and 25 hemadsorption units (HAU) of ASFV Armenia 2008, when delivered in 2 mL of splenic suspension, caused infection in wild boar, with administration of 3 HAU infecting only the weakest animals [31]. Niederwerder et al. (2019) estimated, based on a sequential adaptive experiment using ASF strain Georgia 2007, that ID50 was 10^1.0 and 10^6.8 TCID50 (equivalent to HAD50 [32]) for liquid and feed respectively [33]. Based on these recent findings, we modeled the coefficient *c* in Model 6 as 10^Uuniform (-4.155, -1.558): the dose response model with coefficient *c* of this distribution assumes any doses between 25 HADs and 10^4 HADs as a median infectious dose with an equal probability (Fig 4).

### Model implementation

The model was developed in @Risk Version 7.6 (Palisade, Ithaca, New York) within Microsoft® Excel 2013, and was run with 10000 iterations using Latin Hybercube sampling for each simulation. Results of model outputs are presented as: Mean (5th percentile, 95th percentile).

### Sensitivity analysis

To assess the effect of uncertainty in the current model, sensitivity analysis was performed using Spearman’s correlation coefficient to rank all model input parameters according to their contributions to the variance of model output *P_introduction*.

### Scenario analysis

Scenario analysis was performed to assess the effect of changes in selected input variables summarized in Table 2. A two-way scenario analysis was also performed to assess the effect of simultaneous changes of *P_ecofeed heat-treated* and *P_swill heat-treated on farm*.

**Table 2:** List of scenarios that were tested in scenario analysis and the modified input variables under each scenario.

Input variable	Scenario	Value	Comments
*N_affected farms*	1	56 (baseline)	To assess the effect of increased number of outbreaks in China
	2	2 fold increase	
	3	3 fold increase	
	4	5 fold increase	
*P_illegal import*	1	1%	To assess the effect of an increased probability of illegal importation of pork products by air travelers from China
	2	Beta (8, 242) (baseline)	
	3	5%	
	4	10%	
*P_swill heat-treated on farm*	1	Beta (68, 3) (baseline)	To assess the effect of an decreased proportion of farms practicing heat-treatment before feeding swill to pigs
	2	75%	
	3	90%	
	4	99%	
*P_ecofeed heat-treated*	1	100% (baseline)	To assess the effect of some proportion of Ecofeed not properly heat-treated
	2	99%	
	3	95%	
	4	90%	

## Results

### Risk quantification

**Fig 5:**
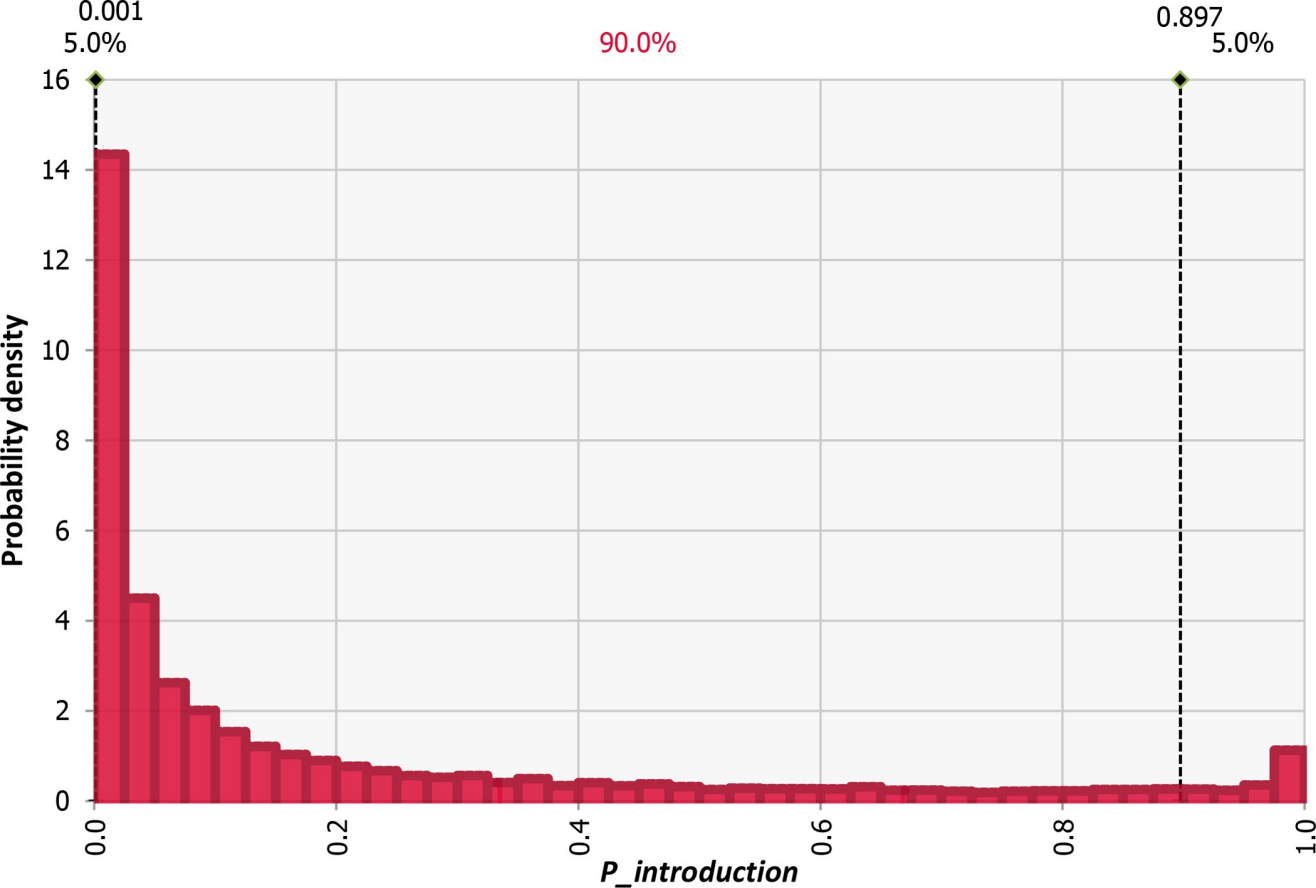
Distribution of the probability of at least one pig becoming infected by consuming non heat-heated swill originating from pork products illegally imported by air travelers from China (*P_introduction*).

The probability of at least one pig becoming infected with ASFV in one year was predicted to be 0.20 (90% PI: 0.00–0.90) (Fig 5).

### Sensitivity analysis

**Fig 6:**
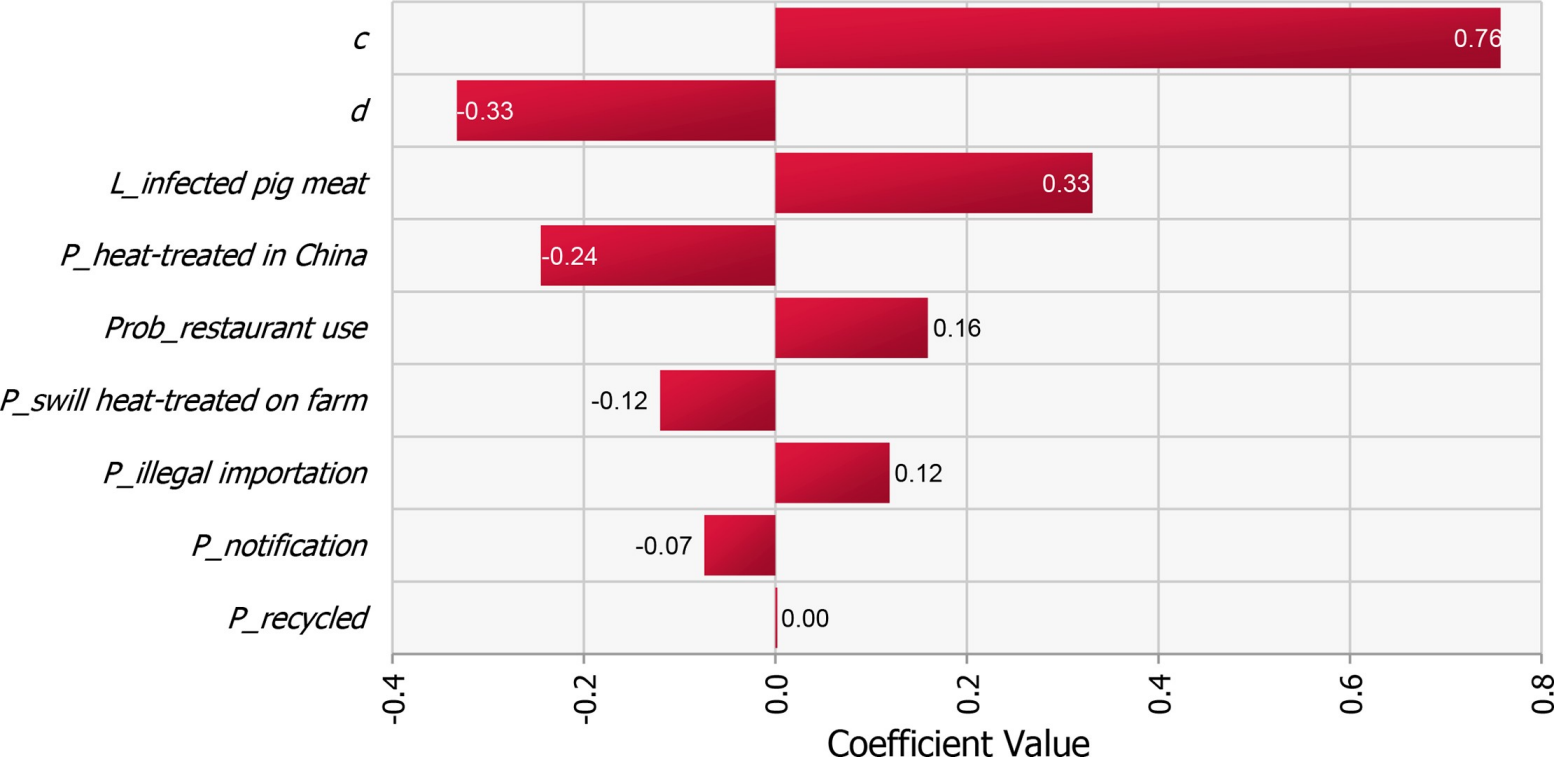
Tornado graph illustrating the results of sensitivity analysis. All model input parameters are ranked by Spearman’s correlation coefficient according to their contribution to the variance of model output *P_introduction*. The nine most correlated input parameters are shown.

The results of the sensitivity analysis are shown in Fig 6. The most correlated parameters were the dose response coefficient (*c*), followed by the probability that infected pigs die on affected farms (*d*), the infectious load of ASFV in an infected pig (*L_infected pork*), the probability that the pork product is heat-treated in China (*P_heat-treated in China*) and the probability that the infected pork product illegally imported is used in restaurants (*Prob_restaurant use*).

### Scenario analysis

**Fig 7:**
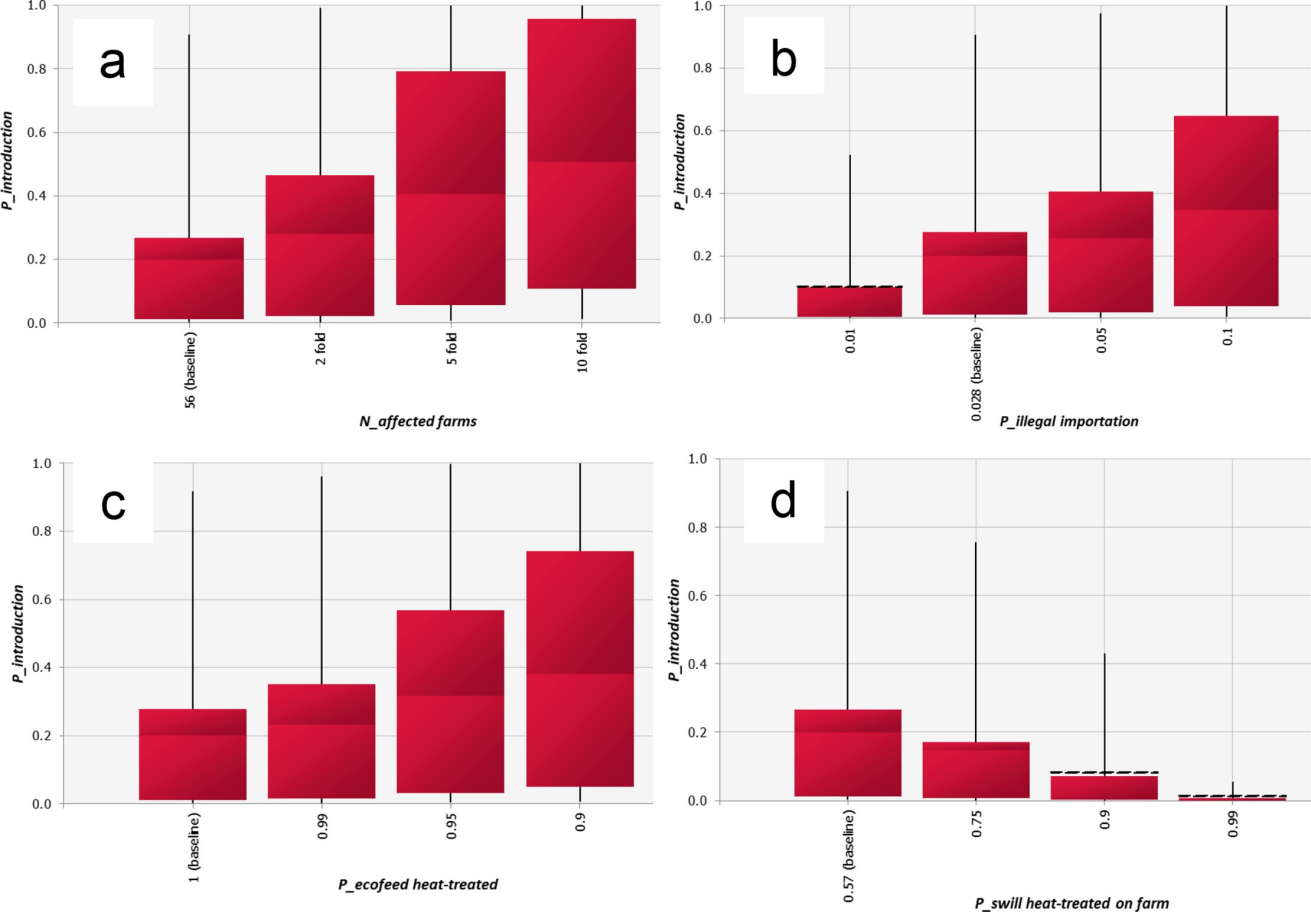
Scenario analysis depicting the effects of tested scenarios on the probability of at least one pig becoming infected by consuming untreated swill originating from pork products illegally imported by air travelers from China (*P_introduction*). For each box-whisker plot, the line in the box indicates the mean; the length of the box indicates the inter-quartile range; the whiskers indicate the 5th percentile and the 95th percentile respectively. For b and d, fixed values of 0.028 and 0.57 were used as the baseline respectively.

The effect of different scenarios on the probability of ASF introduction (*P_introduction*) was investigated and results are shown in Fig 7. From this analysis, it was concluded that an increase in the number of outbreaks in China will increase the risk of ASF introduction, with *P_introduction* rising to 0.51 (0.01–1.00) if the number of outbreaks increase ten fold (Fig 7A).

*P_introduction* will decrease to 0.10 (0.00–0.52) with the *P_illegal importation* of 1%. Contrarily, *P_introducction* will increase to 0.35 (0.04–1.00) with the *P_illegal importation* of 10% (Fig 7B).

*P_introduction* will substantially increase if a proportion of Ecofeed is not properly heat-treated during the production process: *P_introduction* will increase to 0.23 (0.00–0.96), 0.31 (0.00–1.00) and 0.38 (0.01–1.00) if 1%, 5% and 10% of Ecofeed is not properly heat-treated respectively (Fig 7C).

*P_introduction* will decrease to 0.15 (0.00–0.76), 0.08 (0.00–0.43) and 0.01 (0.00–0.05) as the proportion of swill feeding pig farms that apply heat-treatment increases to 75%, 90% and 99% respectively (Fig 7D).

**Fig 8:**
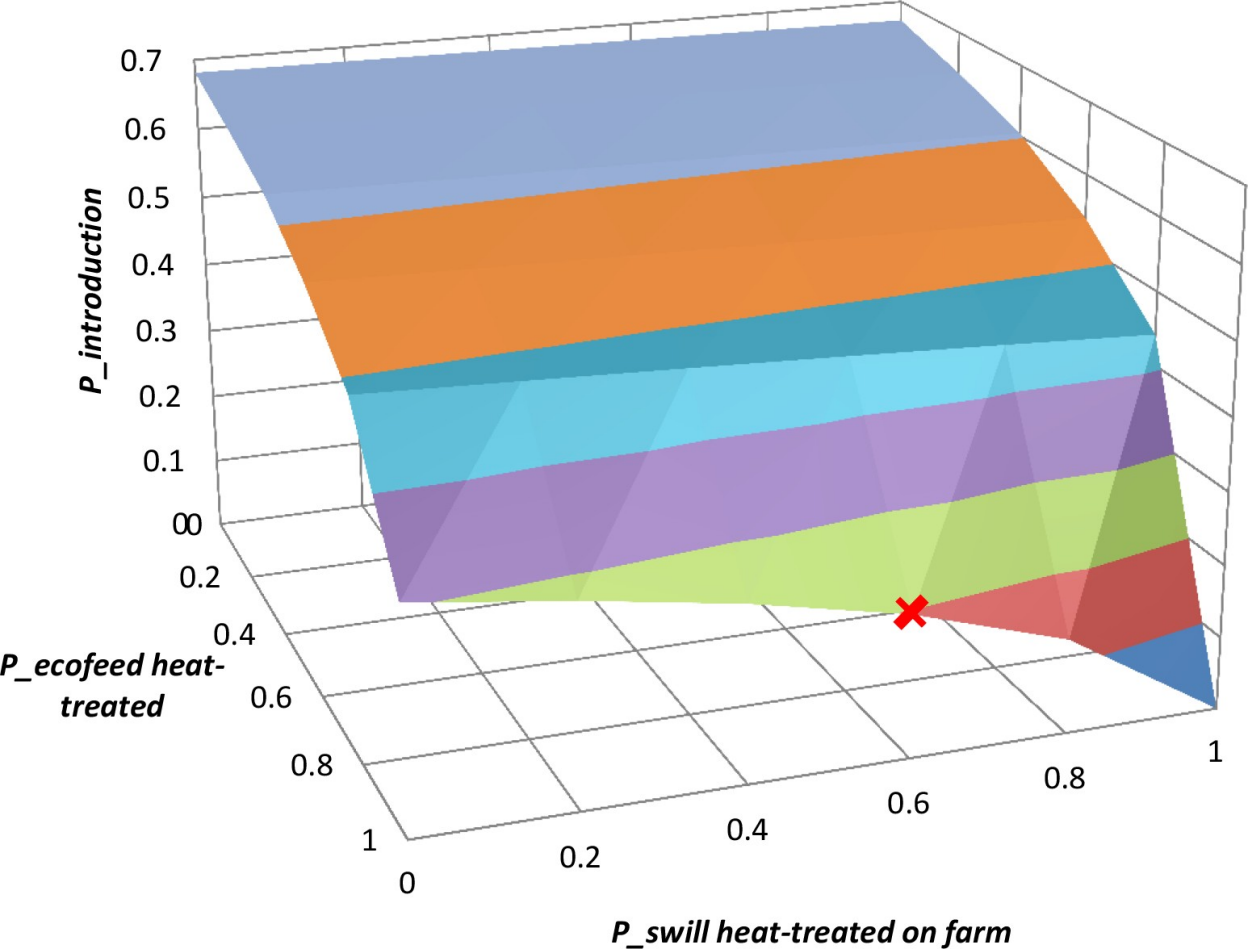
Two-way scenario analysis depicting the effects of *P_ecofeed heat-treated* and *P_swill heat-treated on farm* on the mean value of *P_introduction*. The red X indicates the mean value of P*_introduction* with *P_ecofeed heat-treaed* and *P_swill heat-treated on farm* taking the baseline values (1.0 and Beta (8, 6) respectively).

The result of two-way scenario analysis assessing the effect of simultaneous changes of *P_ecofeed heat-treated* and *P_swill heat-treated on farm* is shown in Fig 8. The change of *P_ecofeed* from the baseline value of 1 has a more significant effect on *P_introduction* than change of *P_swill heat-treated on farm* from its baseline value.

## Discussion

The risk of ASF introduction into Japan through the illegal importation of pork products by air travelers from China and swill feeding identified in this study is relatively high and accompanied with a large degree of uncertainty. The uncertainty associated with the risk of introduction is mainly due to the dose response relation of ASFV, followed by the probability of an infected pig dying on affected farms, loading of ASVF in an infected pig, probability of an illegally imported pork product being heat-treated in China and used in restaurants (Fig 6), indicating that there is a need to collect more data on these parameters to reduce the uncertainty of prediction.

Illegal importation or smuggling of meat and meat products is a serious issue which could greatly compromise a country’s import regime in preventing the introduction of diseases. Its effect on the risk of entry of ASF and other TADs has been evaluated in various QRA [34–40]. Most recently, Jurado et al. (2019) and Ito et al. (2019) assessed the risk of ASF introduction into the US and Japan respectively through smuggling of pork products in air passenger luggage [36, 37]. They both calculated the risk using two main inputs without considering the exposure assessment, namely, i) number of prohibited swine products of air passengers confiscated at airports and ii) number of air passengers arriving in the US and Japan via international commercial flights.

Although not totally comparable because of the methodological difference, the risk calculated in our study was lower than the risk calculated previously by Ito et al.: they predicted that the annual risk of ASFV introduction from China into Japan via pork product brought in in air passenger’s luggage is 0.67 (95% PI: 0.223–0.999) [37]. This is mostly because that they only considered the release risk in assessing the risk of introduction while in our current model we took account of both release and exposure risk.

The impact of illegal importation of pork products was assessed in the current model by changing the probability of illegal importation. The result indicates that the risk of ASF introduction into Japan would increase 1.8 times if the probability of illegal importation increases from the current level to 10% (Fig 7B). This analysis is essential in highlighting the importance of minimization of illegal imports of pork products by air travelers through awareness raising campaign and use of detector dogs at the airports targeting air travelers visiting Japan from ASF affected countries.

In our baseline model we assumed that infected pork product waste used for the production of Ecofeed presents no risk, because the required heat-treatment condition of 70 degree Celsius for 30 minutes is considered to be sufficient to inactivate ASFV. However, although ASFV is shown to be inactivated by heating at 56°C/70 minutes or 60°C/20 minutes [41], depending on the components that constitute the swill there might be a chance that ASFV is not inactivated even with this heat-treatment condition. Meat and swill cannot be compared because their water/fat content is very different, and with a diversity of materials that can be present in swill, uniform heating may not occur and some materials could potentially protect the virus [42]. The World Organisation for Animal Health (OIE) recommends a higher heat-treatment condition, i.e. 90°C for at least 60 minutes, with continuous stirring; or 121°C for at least 10 minutes at an absolute pressure of 3 bar [43]. The quality management programmes practiced by the Ecofeed plants should be monitored to ensure that heat-treatment conditions prescribed by the guidelines are observed [26]. Depending on the type of food waste used for Ecofeed, the MAFF should even consider amending the guidelines to strengthen the heat-treatment requirements, in line with the recommended procedures for the inactivation of ASFV in swill by the OIE. This should be reiterated as the result of our two-way scenario analysis indicated that even a small change of *P_ecofeed heat-treated* from 1 will result in a more significant rise in *P_production* than a change of *P_swill heat-treated on farm* (Fig 8).

The result of our scenario analysis revealed that with an increased proportion of pig farms practicing heat-treatment of swill before feeding to pigs, the risk of ASF introduction can be effectively reduced. The swill feeding practice in pig farms should be regularly monitored to ensure that consistent heat-treatment procedures are practiced, or this practice should be banned when the *W_release* is high, to protect Japanese pig industry from the introduction of ASF.

In our model, we assumed that infected pork products that are not heat-treated have the same infectious load as that of muscle of an infected pig at slaughter [28], because no data were available regarding the change in infectious load of ASFV in non-heat-treated or improperly heat-treated pork products. With this assumption, our model might be over-estimating the risk of ASF entry into Japan.

With the limited amount of data on some of the input variables, the result of our risk assessment was accompanied with a large degree of uncertainty. Nevertheless, it has given us insight into what measures should be taken to help protect the Japanese pig industry from the introduction of ASF and identified what additional data are needed to conduct risk assessment with a greater degree of certainty.

In this study, only travelers from China were considered. The risk posed by travelers from other ASF infected countries both in Asia and other regions was not considered. Considering the number of travelers arriving in Japan from China compared to other countries, travelers from China pose the highest risk as suggested in our previous study [15]. However, as ASF has been spreading to other Asian countries and other regions, it would be worthwhile conducting the risk assessment of ASF entry to Japan from other countries with additional information.

## Supporting information

S1 File ASF risk assessment model. (XLSX)
